# Tai Chi for cancer survivors: A systematic review toward consensus‐based guidelines

**DOI:** 10.1002/cam4.4273

**Published:** 2021-09-17

**Authors:** Lin Yang, Kerri Winters‐Stone, Benny Rana, Chao Cao, Linda E. Carlson, Kerry S. Courneya, Christine M. Friedenreich, Kathryn H. Schmitz

**Affiliations:** ^1^ Department of Cancer Epidemiology and Prevention Research Alberta Health Services Calgary Canada; ^2^ Departments of Oncology University of Calgary Calgary Canada; ^3^ Departments of Community Health Sciences University of Calgary Calgary Canada; ^4^ School of Nursing and Knight Cancer Institute Oregon Health & Science University Portland Oregon USA; ^5^ Program in Physical Therapy Washington University School of Medicine St Louis Missouri USA; ^6^ Department of Psychosocial Oncology Alberta Health Services Calgary Canada; ^7^ Faculty of Kinesiology, Sport, and Recreation University of Alberta Edmonton Canada; ^8^ Penn State Cancer Institute Penn State College of Medicine Hershey Pennsylvania USA

**Keywords:** cancer survivor, cancer treatment, exercise, systemic review, Tai Chi

## Abstract

To manage acute, long‐term, and late effects of cancer, current guidelines recommend moderate‐to‐vigorous intensity aerobic and resistance exercise. Unfortunately, not all cancer survivors are able or willing to perform higher intensity exercise during difficult cancer treatments or because of other existing health conditions. Tai Chi is an equipment‐free, multicomponent mind–body exercise performed at light‐to‐moderate intensity that may provide a more feasible alternative to traditional exercise programs for some cancer survivors. This systematic review evaluated the therapeutic efficacy of Tai Chi across the cancer care continuum. We searched MEDLINE/PubMed, Embase, SCOPUS, and CINAHL databases for interventional studies from inception to 18 September 2020. Controlled trials of the effects of Tai Chi training on patient‐reported and objectively measured outcomes in cancer survivors were included. Study quality was determined by the RoB 2 tool, and effect estimates were evaluated using the Best Evidence Synthesis approach. Twenty‐six reports from 14 trials (one non‐randomized controlled trial) conducted during (*n* = 5) and after treatment (after surgery: *n* = 2; after other treatments: *n* = 7) were included. Low‐level evidence emerged to support the benefits of 40–60 min of thrice‐weekly supervised Tai Chi for 8–12 weeks to improve fatigue and sleep quality in cancer survivors. These findings need to be confirmed in larger trials and tested for scaling‐up potential. Insufficient evidence was available to evaluate the effects of Tai Chi on other cancer‐related outcomes. Future research should examine whether Tai Chi training can improve a broader range of cancer outcomes including during the pre‐treatment and end of life phases.

## Graphical abstract

Tai Chi training may improve fatigue and sleep quality in cancer survivors. Future research should examine a broader range of outcomes, particularly during difficult cancer treatments and for those with significant comorbidities.

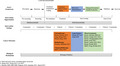

## 1 INTRODUCTION

To improve acute, long‐term, and late effects of cancer and its treatments, the American College of Sports Medicine (ACSM) recommends cancer survivors engage in moderate‐to‐vigorous intensity aerobic exercise at least thrice per week for at least 30 min, and/or resistance training at least twice per week for at least 8–12 weeks.^1^ The ACSM guidelines noted the potential value of, but the lack of evidence for, exercise modes other than conventional aerobic and resistance training, such as yoga and Tai Chi.^2^ Indeed, difficult treatments and pre‐existing health conditions may limit the engagement of older and/or deconditioned individuals in conventional moderate‐to‐vigorous exercise.^3^ Low‐impact and lower intensity exercises like yoga and Tai Chi may provide additional opportunities for cancer survivors to be active.^1^

Tai Chi (or Tai Chi Chuan, Taiji, Taijiquan) is an exercise modality that originated in China thousands of years ago that combines Chinese philosophy with martial and healing arts.^4^ The modern forms of Tai Chi evolved from the 1700s and led to several styles (e.g., Chen‐, Yang‐, Wú‐, Wǔ‐, and Sun‐Style), which all share similar principles.^5^ The practice of Tai Chi is characterized by slow, flowing physical movements that are coordinated with diaphragmatic breathing, musculoskeletal stretching and relaxation, kinesthetic body awareness (mental focus on muscle and movement sense), and a meditative state of mind.^6^ Empirical studies have reported the energy cost of Tai Chi to range from 2.6 to 6.5 Metabolic Equivalent (MET),^7^ which is classified as light‐to‐moderate intensity.^8^

To date, many studies have demonstrated clinically meaningful improvements associated with Tai Chi in individuals with chronic conditions.^9^ Several reviews have summarized the benefits of Tai Chi specific to cancer; however, these reviews were either limited to breast cancer,^10^, ^11^, ^12^, ^13^, ^14^, ^15^ or focused on the general quality of life and selected symptoms.^16^, ^17^, ^18^, ^19^ Only one review attempted to characterize the Tai Chi curricula/protocols.^20^ More importantly, moving research into practice in oncology requires a systematic and comprehensive road mapping of research evidence in relation to the specific phase of the cancer care continuum, which has been previously absent.^20^, ^21^, ^22^, ^23^

We aim to conduct a systematic review of the effects of Tai Chi in cancer survivors at all phases of the cancer continuum. We follow a logic model^24^ adapted from the previously published framework PACC (Physical Activity and Cancer Control), an organizational model for examining physical exercise across the cancer continuum,^25^ to create a roadmap for Tai Chi research in cancer care. This review also aims to identify knowledge gaps to inform future research in areas with limited evidence and inform clinical practice in areas with sufficient evidence.

## 2 METHOD

### 2.1 Search strategy

This review was registered a priori (CRD42020210365) and executed following the PRISMA statement guideline. We searched electronic databases MEDLINE/PubMed, Embase, SCOPUS, and CINAHL from inception to 18 September 2020 with the following search keys: (malignant OR neoplasia OR tumor OR tumor OR carcinoma OR cancer) AND (taiji OR “tai ji” OR taichi OR "tai chi" OR qigong OR "qi gong"). We additionally hand‐searched the reference lists of eligible articles and other narrative overviews of systematic reviews/meta‐analyses.

### 2.2 Study selection and inclusion criteria

We included studies that examined Tai Chi interventions among cancer survivors. The specific inclusion criteria were: (1) including a control or comparison group; (2) an outcome assessment (physical, psychosocial, or clinical outcomes) reported; (3) pre‐post measures on study outcomes reported before and after the intervention; and (4) outcomes reported as either an odds ratio, relative risk, hazard ratio, or continuous data with mean ± standard deviation or 95% confidence intervals. We set no restrictions on the language of the studies published provided an English abstract was available.

One author (LY) removed irrelevant references by screening titles and abstracts, with 5% of those excluded verified by another author (CC). The remaining abstracts were assessed by one author (LY) against inclusion criteria, with all exclusion verified by another author (CC). Next, one author (LY) assessed the full texts of all remaining studies for final inclusion, verified by another author (CC). Discrepancies were resolved in discussion with a third author (BR).

### 2.3 Data extraction

For each included study, two authors (BR and LY) extracted the data, verified by a third author (CC). One author (LY) summarized the study validity, verified by another author (CC). Any discrepancies were resolved by discussion. The following data were extracted for each study: first author name, year of publication, journal, country, funding; primary aim, cancer site and stage, treatment modality, timing of the intervention in respect to treatment phase, study design, ethical approval; the procedures for defining, recruiting, and sampling from the intervention and control groups; the characteristics and sample size of the study population; author description of Tai Chi; the theoretical basis, content, and dose of the intervention and its integrity of delivery; the frequency and duration of follow‐up; the definition and measures of outcomes; and the reference group in statistical modeling, results of statistical tests reported; subgroup analyses, and evidence relating to effects on health outcomes.

### 2.4 Data synthesis

Given the heterogeneity of the types of cancer, treatment modality, and outcomes, meta‐analysis was neither feasible nor appropriate. Hence, data were synthesized following the reporting guidelines of synthesis without meta‐analyses (SWiM) for each phase of the cancer care continuum (Table S1).^26^

We summarized each trial according to effect estimates for each outcome compared to the control group. We presented results in terms of the direction of the effect estimation on the outcome examined wherever possible and grouped outcomes by the timing of the intervention in accordance with the Framework PACC in a quantitative narrative synthesis.

We focused on summarizing outcomes identified as the most common acute, long‐term, and late effects of cancer and its treatments as framed in the recently published exercise guidelines for cancer survivors (anxiety, bone health, cardiotoxicity, chemotherapy‐induced peripheral neuropathy, cognitive function, depressive symptoms, falls, fatigue, health‐related quality of life, lymphedema, nausea, pain, physical function, sexual function, sleep, and treatment tolerance).^1^ Other outcomes were summarized separately.

### 2.5 Methodological quality and evidence synthesis

We assessed the methodological quality of the included studies using the RoB 2 tool (revised Cochrane tool for Risk of Bias in randomized trials).^27^ The RoB 2 tool evaluates the following domains in randomized controlled trials (RCTs): bias arising from the randomization process, bias due to deviations from intended interventions, bias due to missing outcome data, bias in the measurement of the outcome, bias in the selection of the reported results, and summarized the overall risk of bias to low risk (high quality), some concern (medium quality), and high risk (low quality).

Alternatively to meta‐analysis, trials that used nonactive controls were summarized using the “Best Evidence Synthesis” approach.^28^, ^29^ Drawing on study quality (as determined by the RoB tool) and the direction of effect estimations, this system evaluates and grades evidence along with five levels: (i) strong evidence: 2 high‐quality studies in agreement about an outcome; (ii) moderate evidence: 1 high‐quality +1 medium quality, or 3 medium‐quality studies in agreement; (iii) low evidence: 1 high quality, or 2 medium quality, or 3 low studies in agreement; (iv) no evidence: 1 medium quality study, or <3 studies of low‐quality studies, or any number of no quality studies in agreement, or no studies at all; and (v) conflicting evidence: contrasting findings of low to strong evidence among studies.

## 3 RESULTS

A total of 864 references were retrieved from the initial search for title and abstract screening. Among the 53 full texts that were screened, 26 met inclusion criteria. The screening procedure and reasons for exclusion are illustrated in Figure S1 and Table S2.

### 3.1 Characteristics of the included studies

**FIGURE 1:**
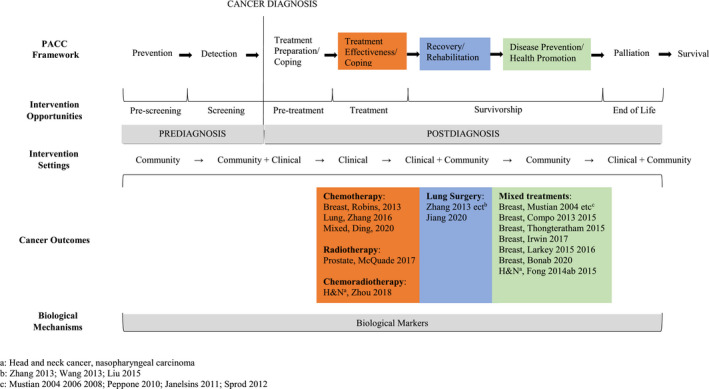
Adapted framework PACC (Physical Activity and Cancer Control, Courneya & Friedenreich 2007) for Tai Chi research in cancer care

Among the 26 studies, findings from 14 Tai Chi trials (13 RCTs and 1 NRCT) were reported (Figure 1), including six conducted in the United States,^30^, ^31^, ^32^, ^33^, ^34^, ^35^, ^36^, ^37^, ^38^, ^39^, ^40^, ^41^, ^42^, ^43^ six conducted in China^44^, ^45^, ^46^, ^47^, ^48^, ^49^, ^50^ (one in Hong Kong special administrative region of China),^51^, ^52^, ^53^ and one each conducted in Thailand^54^ and Iran,^55^ respectively. The sample sizes ranged from nine to 57 in each group.

### 3.2 Characteristics of the study participants

We summarized the study characteristics in Table S3. Seven trials were conducted in survivors of breast cancer,^30^, ^32^, ^33^, ^34^, ^35^, ^36^, ^37^, ^38^, ^39^, ^40^, ^41^, ^42^, ^43^, ^54^, ^55^ three in lung cancer,^47^, ^48^, ^49^, ^50^ two in head & neck cancer,^46^, ^51^, ^52^, ^53^ one in prostate cancer,^31^ and one in mixed cancer.^45^ The disease stage varied from stage 0 to IV with different treatments including chemotherapy, radiotherapy, chemoradiotherapy, and surgery.

### 3.3 Characteristics of the Tai Chi interventions

We summarized the intervention characteristics in Table S4. A variety of Tai Chi forms were used, including 24‐form Yang style Tai Chi (*n* = 4),^45^, ^46^, ^47^, ^48^, ^49^ adapted Yang style Tai Chi (*n* = 2),^31^, ^32^, ^33^, ^34^, ^35^, ^36^, ^37^ 8‐form Tai Chi (*n* = 2),^30^, ^44^ 18‐form Tai Chi Qi Gong (*n* = 2),^51^, ^52^, ^53^, ^54^ Tai Chi Chih (*n* = 2),^38^, ^39^, ^40^, ^41^ Qigong/Tai Chi easy (*n* = 1),^42^, ^43^ and adapted 20‐form Tai Chi (*n* = 1).^55^ Despite the differences, Tai Chi is commonly described as a “mind–body” exercise that involves “physical exercise” (or “movement”), “breathing,” and “meditation” (or “mindfulness”), hence a “meditative movement” (Table S4).

The intervention dose ranged from 90 min per session per week to 60 min per session at five times per week, with the most common dose being thrice‐weekly supervised sessions of 60 min. Although the intervention duration varied from 7–8 weeks to 6 months, most (9/14) studies had an intervention of 12 weeks, with each the rest of 7–8 weeks, 10 weeks, 16 weeks, 19 weeks, and 6 months.

### 3.4 Characteristics of the comparison groups

Five trials used usual care as the control group (nonactive control),^46^, ^47^, ^48^, ^49^, ^51^, ^52^, ^53^, ^54^, ^55^ whereas seven used active controls including other types of exercise,^42^, ^43^, ^44^, ^45^, ^50^ psychosocial support,^32^, ^33^, ^34^, ^35^, ^36^, ^37^ health education,^38^, ^39^ and cognitive behavioral therapy for insomnia.^40^, ^41^ In addition, two trials used a three‐armed design, which included a Tai Chi group, a nonactive control group (usual care^30^ and waitlist control,^31^ respectively), and an active control group (spiritual growth^30^ and other exercise,^31^ respectively).

### 3.5 Outcomes assessed

Several common acute, long‐term, and late effects of cancer that evaluated in the recent exercise guidelines for cancer survivors^1^ were examined in Tai Chi interventions, including anxiety (*n* = 1), depressive symptoms (*n* = 3), fatigue (*n* = 6), health‐related quality of life (*n* = 6), physical function (*n* = 5), bone health (*n* = 1), sleep (*n* = 5), cognitive function (*n* = 1), and pain (*n* = 2) (Tables S5a and 5b). The instruments used to assess these outcomes are summarized in Table S6.

We summarized other outcomes assessed in Tai Chi trials in Table S7 including aerobic capacity,^32^, ^33^, ^34^, ^35^, ^36^, ^37^, ^51^, ^52^, ^53^ and biological markers of hormone,^32^, ^33^, ^34^, ^35^, ^36^, ^37^, ^38^, ^39^, ^47^, ^48^, ^49^, ^54^ neuroendocrine,^30^ inflammation,^32^, ^33^, ^34^, ^35^, ^36^, ^37^, ^38^, ^39^, ^40^, ^41^, ^50^ and immunity.^30^, ^47^, ^48^, ^49^

### 3.6 Summary of effects by phase of the cancer continuum

In accordance with the PACC framework, five RCTs^30^, ^31^, ^44^, ^45^, ^46^ (three used non‐active controls^30^, ^31^, ^46^) were conducted during cancer treatment including chemotherapy (breast, lung, and mixed cancer); radiotherapy (prostate cancer); and chemoradiotherapy (nasopharyngeal carcinoma). Tai Chi trials conducted during cancer treatment found mixed findings in fatigue^31^, ^44^, ^46^ and health‐related quality of life,^30^, ^45^ with one study each reported null results in depressive symptoms^30^ and pain,^45^ but improved sleep.^31^

Two RCTs^47^, ^48^, ^49^, ^50^ (one used non‐active control^47^, ^48^, ^49^) were conducted among post‐surgery non‐small cell lung cancer survivors. One trial^47^, ^48^, ^49^ was designed to evaluate the biological mechanisms and reported no data on common acute, long‐term, and late effects of cancer. Another trial^50^ found that Tai Chi improved patient‐reported pain comparing to physical exercise as control.

Six RCTs^32^, ^33^, ^34^, ^35^, ^36^, ^37^, ^38^, ^39^, ^40^, ^41^, ^42^, ^43^, ^54^, ^55^ (two used non‐active controls^54^, ^55^) and one NRCT^51^, ^52^, ^53^ (vs. non‐active control) were conducted among cancer survivors post‐non‐surgical treatments: six in breast and one in nasopharyngeal carcinoma. Several outcomes were assessed in at least two trials, showing consistent improvement in fatigue,^32^, ^33^, ^34^, ^35^, ^36^, ^37^, ^42^, ^43^, ^54^ and mixed findings in depressive symptoms,^42^, ^43^, ^55^ health‐related quality of life,^32^, ^33^, ^34^, ^35^, ^36^, ^37^, ^38^, ^39^, ^51^, ^52^, ^53^, ^54^ physical function,^32^, ^33^, ^34^, ^35^, ^36^, ^37^, ^38^, ^39^, ^42^, ^43^, ^51^, ^52^, ^53^, ^55^ and sleep.^40^, ^41^, ^42^, ^43^, ^51^, ^52^, ^53^, ^55^ In addition, each of anxiety,^55^ bone health,^32^, ^33^, ^34^, ^35^, ^36^, ^37^ and cognitive function^42^, ^43^ were assessed in one trial.

A detailed description of the study design and findings for each trial are summarized in the Data S1. Notably, two trials that employed 6‐minute walk test found that 12‐week Tai Chi training improved aerobic capacity compared to psychosocial support for post‐treatment breast cancer survivors^32^, ^33^, ^34^, ^35^, ^36^, ^37^ and to usual care for post‐treatment head and neck cancer survivors,^51^, ^52^, ^53^ respectively.

### 3.7 Methodological quality and evidence synthesis

The quality of the included 26 studies is summarized in Table S8. Following the RoB 2 tool, most of the studies were ranked to have some concern (medium quality, *n* = 14) or high risk of bias (low quality, *n* = 9), with three studies ranked at low risk (high quality). The main reason for ranking the study quality down was the unavailability of a study protocol (no link to ClinicalTrial.gov provided by the authors, nor study protocol published and cited by the authors), thus with unknown bias in selection of the reported outcomes. Other reasons to rank the study quality down were missing outcome data, poor measurement of the outcome, and using a non‐randomized design in one trial.

Following the Best Evidence Synthesis, the study quality and the direction of effect estimations are summarized in Table 1, restricting to studies that employed non‐active controls (*n* = 6).^30^, ^31^, ^46^, ^51^, ^52^, ^53^, ^54^, ^55^ Low‐level evidence (two medium quality studies in agreement) emerged for Tai Chi training to improve fatigue and sleep, and no evidence emerged for other outcomes deemed important for cancer survivors primarily due to lack of high‐quality studies.

**TABLE 1:** Best Evidence Synthesis^a^ from Tai Chi interventions that employed a non‐active control group

Evidence level from exercise interventions^b^	Current evidence from Tai Chi interventions First author, publication year, study quality (Low, Medium, and High), and effect estimates (↓ ↔ ↑)			Evidence Level
Strong				
Anxiety	Bonab 2020: Medium ↑			No evidence
Depressive symptoms	Robins 2013^a^: Medium ↔	Bonab 2020: Medium ↑		No evidence
Fatigue	McQuade 2017^a^: Medium ↔	Zhou 2018: Medium ↑	Thongteratham 2015: Medium ↑	Low
Health‐related quality of life	Robins 2013^a^: Medium ↔	Thongteratham 2015: Medium ↑	Fong 2014ab 2015: Low ↔	No evidence
Lymphedema				
Physical function	Bonab 2020: Medium ↑	Fong 2014ab 2015: Low ↑		No evidence
Moderate				
Bone health				No evidence
Sleep	McQuade 2017^a^: Medium ↑	Bonab 2020: Medium ↑	Fong 2014ab: Low ↑	Low
Insufficient				No evidence
Cardiotoxicity				No evidence
Chemotherapy‐induced peripheral neuropathy				No evidence
Cognitive function				No evidence
Falls				No evidence
Nausea				No evidence
Pain				No evidence
Sexual function				No evidence
Treatment tolerance				No evidence

^a^ Best evidence synthesis evidence level: Strong evidence: 2 high‐quality studies in agreement about an outcome; Moderate:1 high‐quality +1 medium quality, or 3 medium‐quality studies in agreement; Low: 1 high quality, or 2 medium quality, or 3 low studies in agreement; No evidence: 1 medium quality study, or <3 studies of low quality studies, or any number of no quality studies in agreement, or no studies at all; Conflicting: contrasting findings of low to strong evidence among studies.

^b^ In accordance with Compbell et al, Medicine & Sciences in Sports & Exercise 2019.

## 4 DISCUSSION

This review provides a comprehensive overview of studies that investigated the effects of Tai Chi training after a cancer diagnosis. Twenty‐six studies were identified, reporting findings from 13 RCTs and 1 NRCT that were conducted during cancer treatment (*n* = 5), post‐surgical rehabilitation (*n* = 2), and post‐mixed treatments (*n* = 7), respectively. These studies generally described Tai Chi by three key components: physical movement/exercise, breathing, and meditation/mindfulness, namely meditative movement. Low‐level evidence (i.e., two medium quality studies in agreement) emerged to support the benefits of 40–60 min of thrice‐weekly supervised Tai Chi training for 8–12 weeks to improve fatigue and sleep quality in cancer survivors during and after treatments. The small number of studies limited the evaluation of other important outcomes.

The present review is the first to comprehensively evaluate the evidence base for Tai Chi interventions in cancer care. We identified emerging evidence on Tai Chi training to improve fatigue and sleep, which agreed with previous reviews that focused on these specific outcomes.^13^, ^17^, ^18^, ^19^ Although the evidence level was deemed low, it is promising to consider the small number of trials (*n* = 3) that were evaluated through a well‐defined and conservative evidence synthesis approach. Given the small number of trials available, it is not possible to draw firm conclusions on other outcomes at this time.

The biological mechanisms through which Tai Chi may improve fatigue and sleep are not entirely clear. Tai Chi has been proposed to improve sleep by reducing sympathetic arousal and inflammation.^41^ Tai Chi, or exercise in general, could possibly improve sleep by regulating the circadian rhythm.^56^ With respect to the etiology of treatment‐related fatigue, inflammation, altered immune response, and mitochondrial dysfunction are among the leading hypothesized biological mechanisms.^57^ Tai Chi may have a direct positive impact on immune function and inflammation (Table S7) to reduce fatigue, and it may also indirectly affect these biological pathways through improving cardiorespiratory fitness.^58^, ^59^ We found that two Tai Chi interventions reported improved aerobic capacity in post‐treatment breast and head & neck cancer survivors, respectively, as assessed by the 6‐minute walk test, the best compromise between test duration and ability to discriminate levels of cardiorespiratory fitness.^60^ Despite being considered as a low metabolic demand exercise, Tai Chi may improve cardiorespiratory fitness. Its upper extremity movements typically involve thoracic expansion and stretching, which may strengthen the respiratory muscle.^61^ Additionally, diaphragmatic breathing techniques in Tai Chi may reshape the breathing pattern to reduce the frequency of breath and keep the airways open longer^62^ and activate respiratory muscles.^63^ These changes may be associated with improved cardiorespiratory fitness.^64^ In other patient populations, our previous umbrella review showed strong evidence supporting Tai Chi training to improve VO_2_max among individuals with coronary heart disease (vs. stretching) and heart failure (vs. medication + exercise), and improve 6‐minute walk in individuals with heart failure (vs. aerobics exercise or walking).^9^ Nevertheless, data are lacking to discern whether Tai Chi can produce the same level of cardiorespiratory benefit as conventional exercise in cancer survivors.

Several gaps in evidence were identified in this review that merit attention. First, very few studies investigating the role of Tai Chi across the cancer care continuum. To date, no RCT data exists on the potential value of Tai Chi in the pre‐treatment and end of life phases (Figure 1). Second, no Tai Chi trial assessed nor reported the following common cancer specific outcomes: lymphedema, cardiotoxicity, chemotherapy‐induced peripheral neuropathy, falls, nausea, sexual function, or treatment tolerance. There is, however, one NRCT of breast cancer survivors that was excluded during the review process because it assessed the acute effect of a single bout of Tai Chi exercise.^65^ This trial reported reduced circumference of the affected upper arm, elbow, forearm, and wrist before and after a 6‐minute 18‐form Tai Chi exercise among 11 breast cancer survivors comparing to 12 breast cancer survivor controls.^65^ It is also worth noting that Tai Chi is among the interventions with the strongest efficacy for fall prevention in older adults.^66^ Particularly, Tai Chi may prevent falls by improving chemotherapy‐induced neuropathy and/or vestibular dysfunction in cancer survivors, which is being investigated in an ongoing RCT.^67^ Until these trials are completed, whether Tai Chi is a viable exercise option for cancer survivors to manage these common acute, long‐term, and late effects of cancer and its treatments is virtually unknown.

Third, no Tai Chi trials measured tumor‐specific or disease prognostic markers that reflect treatment efficacy outcomes. This knowledge gap is not limited to Tai Chi but generally lacking in conventional exercise interventions.^68^ Nevertheless, several studies included in the review measured and reported some data on inflammatory and immunological markers among cancer survivors. The certainty of these findings is hampered by their study quality but provides preliminary evidence to further investigate whether Tai Chi training may improve cancer prognosis through these biological pathways.

Fourth, the review studies used mixed control groups. Therefore, comparative evidence on the effects of Tai Chi training as compared to standard care is limited. Even among studies that used active controls, no study compared Tai Chi to conventional exercise training with a clear definition of frequency, intensity, duration, and type.

Future studies are needed to address these identified knowledge gaps. In addition, the burden of comorbidity in cancer survivors is increasing, particularly with aging.^69^ Clearly, the number of comorbidities may pose physical barriers to engage in high‐impact, high‐intensity activity, which may in part explain why the benefit of converntional exercise is understudied in this population. In a comprehensive systematic review of 600 RCTs testing behavioral and/or psychosocial interventions, cancer survivors with comorbidity were excluded from 73.3% of exercise trials reviewed.^70^  This exclusion is critical because despite the potential benefits of prescribing exercise as medicine for cancer survivors,^71^ evidence on the feasibility and efficacy of exercise in those with comorbidities is limited.

One particular challenge of conducting Tai Chi interventions is the need for experienced instructors and the perceived complexity of Tai Chi training, of which transition between moves was viewed as the most difficult component of ancient Tai Chi.^72^  Nevertheless, the recent development of several simplified, yet effective Tai Chi curriculums,^72^, ^73^ and the development of multi‐media technology to deliver by mobile intervention^74^  may be adopted to overcome these barriers. The low metabolic demands and equipment‐free nature of Tai Chi makes it suitable for cancer survivors who may experience difficulties with adherence to conventional exercise programs (or as part of the rehabilitation program) and overcome accessibility barriers.^3^ Nevertheless, the scaling‐up potential of Tai Chi requires standardization of Tai Chi protocols with a clear definition of the physical movement, breathing, and mediation components.

The evidence base supporting the efficacy of Tai Chi for outcomes relevant to cancer survivors is promising. Definitive trials are needed for outcomes for which evidence is considered low but emerging (i.e., fatigue and sleep quality). Ideally, these studies would be three‐armed trials comparing the current exercise guidelines for cancer survivors, Tai Chi training, and a nonactive control group. These studies should enroll well‐characterized study populations in terms of cancer site, treatment phase and modality, and adequately define the intervention dose.

## 5 CONCLUSION

Fourteen trials identified in this review have examined the effects of Tai Chi training during cancer survivorship. Sample sizes were small, but the findings are promising. Low‐level evidence emerged to support the benefits of 40–60 min of thrice‐weekly supervised Tai Chi training for 8–12 weeks to improve fatigue and sleep quality in cancer survivors. There is insufficient evidence to evaluate other common outcomes. Future studies with rigorous designs are needed to fully understand the role of Tai Chi as a viable exercise mode for cancer survivors, particularly during difficult cancer treatments and for those facing comorbidity challenges.

## CONFLICT OF INTEREST

None.

## Supporting information

Supplementary Material

## Data Availability

The data used to support the findings in this review are included within the article and supplementary materials.
