# Supplementary material for: Tai Chi for cancer survivors: A systematic review toward consensus‐based guidelines
Source: Cancer Med. 2021 Sep 17;10(21):7447–56. doi: 10.1002/cam4.4273 (PMC8559497; doi:10.1002/cam4.4273)
Supplement: Supplementary file 1 — Supplementary Material [file CAM4-10-7447-s001.docx]

**Supplementary materials**

**1. Supplementary Table 1.** Synthesis Without Meta-analysis (SWiM) reporting items

**2.** **Supplementary Table 2**. Summary of excluded studies and reason for exclusion (n=27)

**3. Supplementary Table 3.** Characteristics of the included studies on Tai Chi intervention in cancer

**4. Supplementary Table 4.** Tai Chi forms and authors’ interpretation of Tai Chi of the included trials

**5. Supplementary Table 5a.** Evidence level of common acute, long-term, and late effects of cancer reviewed for therapeutic efficacy of exercise that evaluated in Tai Chi interventions

**6. Supplementary Table 5b.** Effect on common acute, long-term, and late effects of cancer of Tai Chi intervention

**7. Supplementary Table 6.** Measures used to assess common acute, long-term, and late effects of cancer of Tai Chi intervention

**8.** **Supplementary Table 7**. Other cancer related outcomes of Tai Chi intervention

**9. Supplementary Table 8**. Summary of study quality assessment based on the Risk of Bias 2 tool

**10. Supplemental Figure 1.** Study flowchart

**11. Detailed description of each included trials of study design and findings**

| **Supplementary Table 1: Synthesis Without Meta-analysis (SWiM) reporting items** | | | |
| --- | --- | --- | --- |
| The citation for the Synthesis Without Meta-analysis explanation and elaboration article is: Campbell M, McKenzie JE, Sowden A, Katikireddi SV, Brennan SE, Ellis S, Hartmann-Boyce J, Ryan R, Shepperd S, Thomas J, Welch V, Thomson H. Synthesis without meta-analysis (SWiM) in systematic reviews: reporting guideline BMJ 2020;368:16890 doi: http://doi.org/10.1136/bmj.16890 | | | |
| SWiM is intended to complement and be used as an extension to PRISMA | | | |
| **SWiM reporting item** | **Item description** | **Page in manuscript where item is reported** | **Others** |
| Item 1: grouping studies for synthesis | 1a) Provide a description of, and rational for, the groups used in the synthesis (e.g., grouping of populations, interventions, outcomes, study design). | Page 3, PACC |  |
|  | 1b) Detail and provide rationale for any changes made subsequent to the protocol in the groups used in the synthesis. |  |  |
| Item 2: describe the standardised metric and transformation methods used | Describe the standardised metric for each outcome. Explain why the metric(s) was chosen, and describe any methods used to transform the intervention effects, as reported in the study, to the standardised metric, citing any methodological guidance consulted. | Page 3, direction of effect |  |
| Item 3: describe the synthesis methods | Describe and justify the methods used to synthesise the effects for each outcome when it was not possible to undertake a meta-analysis of effect estimates. | Page 3, PACC+ACSM guidelines, Best Evidence synthesis |  |
| Item 4: criteria used to prioritise results for summary and synthesis | Where applicable, provide the criteria used, with supporting justification, to select the particular studies, or a particular study, for the main synthesis or to draw conclusions from the synthesis (e.g., based on study design, risk of bias assessments, directness in relation to the review question). | NA |  |
| Item 5: investigation of heterogeneity in reported effects | State the method(s) used to examine heterogeneity in reported effects when it was not possible to do a meta-analysis of effect estimates and its extensions to investigate heterogeneity. | 3 page 3-4 PACC, cancer sites, outcomes |  |
| Item 6: certainty of evidence | Describe the methods used to assess certainty of the synthesis findings. | Page 2, RoS2 |  |
| Item 7: data presentation methods | Describe the graphical and tabular methods used to present the effects (e.g., tables, forest plots, harvest plots).  Specify key study characteristics (e.g., study design, risk of bias) used to order the studies, in the text and any tables or graphs, clearly referencing the studies included. | Tables 1-5, Figure 2 |  |
| Item 8: reporting results | For each comparison and outcome, provide a description of the synthesised findings, and the certainty of the findings. Describe the result in language that is consistent with the question the synthesis addresses, and indicate which studies contribute to the synthesis. | Page 4-5 |  |
| Item 9: limitation of the synthesis | Report the limitations of the synthesis methods used and/or the groupings used in the synthesis and how these affect the conclusions that can be drawn in relation to the original review question. | Page 5 |  |
| PRISMA=Preferred Reporting Items for Systematic Reviews and Meta-Analyses.  *If the information is not provided in the systematic review, give details of where this information is available (e.g., protocol, other published papers (provide citation details), or website (provide the URL)). | | | |

| **Supplementary Table 2: Summary of excluded studies and reason for exclusion (n=27)** | | | |
| --- | --- | --- | --- |
| **First author, year** | **Journal** | **Title** | **Reason for exclusion** |
| Liu, 1987 | Chinese Journal of Modern Developments in Traditional Medicine | [The breathing exercise (qigong) in the treatment of postoperative malignant tumors of the gastrointestinal tract] | Qigong |
| Kerr, 2002 | Culture, Medicine & Psychiatry | Translating "mind-in-body": two models of patient experience underlying a randomized controlled trial of qigong | No outcome |
| Galantino, 2003 | Rehabilitation Oncology | The effects of Tai Chi and walking on fatigue and body mass index in women living with breast cancer: a pilot study | Qigong |
| Lee, 2006 | American Journal of Chinese Medicine | Effects of Chan-Chuang qigong on improving symptom and psychological distress in chemotherapy patients | Qigong |
| Yeh, 2006 | Cancer Nursing | The influences of Chan-Chuang qi-gong therapy on complete blood cell counts in breast cancer patients treated with chemotherapy | Qigong |
| Oh, 2008 | American Journal of Chinese Medicine | Medical Qigong for cancer patients: pilot study of impact on quality of life, side effects of treatment and inflammation | Qigong |
| Oh, 2010 | Annals of Oncology | Impact of medical Qigong on quality of life, fatigue, mood and inflammation in cancer patients: a randomized controlled trial | Qigong |
| Oh, 2012 | Supportive Care in Cancer | Effect of medical Qigong on cognitive function, quality of life, and a biomarker of inflammation in cancer patients: a randomized controlled trial | Qigong |
| Chen, 2013 | Cancer | Qigong improves quality of life in women undergoing radiotherapy for breast cancer: results of a randomized controlled trial | Qigong |
| Loh, 2014 | Asian Pacific Journal of Cancer Prevention | The Kuala Lumpur Qigong trial for women in the cancer survivorship phase-efficacy of a three-arm RCT to improve QOL | Qigong |
| Fong, 2014 | Integrative Cancer Therapies | Effects of qigong exercise on upper limb lymphedema and blood flow in survivors of breast cancer: a pilot study | Single bout Tai Chi |
| Campo, 2014. | Journal of Cancer Survivorship | Levels of fatigue and distress in senior prostate cancer survivors enrolled in a 12-week randomized controlled trial of Qigong | Qigong |
| Chen, 2015 | European Journal of Integrative Medicine | Meditative qigong relieved symptom severity and interference among patients with breast carcinoma receiving chemotherapy | Qigong |
| Huang, 2016 | European Journal of Oncology Nursing | Effects of non-sporting and sporting qigong on frailty and quality of life among breast cancer patients receiving chemotherapy | Qigong |
| Yeh, 2016 | European Journal of Oncology Nursing | A randomized controlled trial of qigong on fatigue and sleep quality for non-Hodgkin's lymphoma patients undergoing chemotherapy | Qigong |
| Han, 2016 | Tianjin journal of traditional Chinese medicine | A clinical study on therapeutic effects of fitness Qigong Baduanjin on pulmonary function and quality of life of post-operative non-small cell lung cancer patients | Qigong |
| Li, 2017 | Chinese Nursing Research | Application of eight-section brocade in postoperative rehabilitation of non-small cell lung cancer patients | Qigong |
| Chuang, 2017 | International Journal of Nursing Studies | A nurse facilitated mind-body interactive exercise (Chan-Chuang qigong) improves the health status of non-Hodgkin lymphoma patients receiving chemotherapy: Randomised controlled trial | Qigong |
| Liu, 2017 | Quality of Life Research | The efficacy of Guolin-Qigong on the body-mind health of Chinese women with breast cancer: a randomized controlled trial | Qigong |
| Vanderbyl, 2017 | Supportive Care in Cancer | A comparison of the effects of medical Qigong and standard exercise therapy on symptoms and quality of life in patients with advanced cancer | Qigong |
| Sowada, 2019 | Clinical Journal of Oncology Nursing | Qigong: Benefits for Survivors Coping With Cancer-Related Fatigue | Case study |
| Ying, 2019 | European Journal of Oncology Nursing | The health effects of Baduanjin exercise (a type of Qigong exercise) in breast cancer survivors: A randomized, controlled, single-blinded trial | Qigong |
| Murley, 2019 | Journal of Holistic Nursing | Influence of Tai Chi on Self-Efficacy, Quality of Life, and Fatigue Among Patients With Cancer Receiving Chemotherapy: A Pilot Study Brief | Group n=3 |
| Myers, 2019 | Supportive Care in Cancer | Qigong intervention for breast cancer survivors with complaints of decreased cognitive function | Qigong |
| Lopez, 2020 | Integrative Cancer Therapies | Effects of Center-Based Delivery of Tai Chi and Qi Gong Group Classes on Self-Reported Symptoms in Cancer Patients and Caregivers | No control group |
| Cheng, 2020 | Journal of Cancer Education | The Health Promoting Mindfulness or Qigong Educational Programs for Beneficial Lifestyle Changes of Cancer Survivors | Qigong |
| Trevino, 2020 | Supportive Care in Cancer | Rapid deployment of virtual mind-body interventions during the COVID-19 outbreak: feasibility, acceptability, and implications for future care | No control group |

| **Supplementary Table 3. Characteristics of the included studies on Tai Chi intervention in cancer** | | | | | | | | | | | | | |
| --- | --- | --- | --- | --- | --- | --- | --- | --- | --- | --- | --- | --- | --- |
| **Study** | **Country** | **Cancer site (stage)** | **Study design**^a^ | **Intervention Providers** | **Session duration** | **Frequency/week** | | **Total duration** | **Control group** | **Period of follow up** | **Sample size** | **Retention** | **Sex (% female)** |
|  |  |  |  |  |  | Supervised | Home |  |  |  |  |  |  |
| **During chemotherapy** | |  |  |  |  |  |  |  |  |  |  |  |  |
| Robins 2013 | US | Breast (early stage) | RCT | Training videotapes/DVDs | 90 min | 1 |  | 10 weeks | (1) spiritual growth  (2) usual care | 70 weeks | 109 | 75% | 100 |
| Zhang 2016 | China | Lung (I-IV) | RCT | Tai Chi instructor | 60 min | 3.5 | | 12 weeks | Low-impact exercise | 12 weeks | 91 | 77% | 92.5 |
| Ding 2020 | China | N.R. (N.R.) | RCT | Full time coach | 60 min | 5 | | 12 weeks | Traditional handgrip exercise | 12 weeks | 98 | 100% | 54.1 |
| **During radiotherapy** | |  |  |  |  |  |  |  |  |  |  |  |  |
| McQuade 2017 | US | Prostate (I-III) | RCT | Tai Chi instructor | 40 min | 3 | daily | 7-8 weeks | (1) light exercise  (2) waitlist control | 12 weeks | 66 | 66% | 0 |
| **During chemoradiotherapy** | | |  |  |  |  |  |  |  |  |  |  |  |
| Zhou 2018 | China | H&N (III-IVa/b)^b^ | RCT | Research team; video | 60 min | 5 |  | 19 weeks | Usual care | 19 weeks | 114 | 73% | 20.2 |
| **Post-surgery** | | |  |  |  |  |  |  |  |  |  |  |  |
| Zhang 2013 ect^c^ | China | Lung (I-IIIb) | RCT | Tai Chi instructor | 60 min | 3 |  | 16 weeks | Usual care | 16 weeks | 32 | 84% | 45.5 |
| Jiang 2020 | China | Lung (N.R.) | RCT | Tai Chi instructor | 60 min | 7 |  | 12 weeks | Common care physical exercise | 70 weeks | 100 | 100% | 44.0 |
| **Post-mixed treatment** | | |  |  |  |  |  |  |  |  |  |  |  |
| Mustian 2004 etc^d^ | US | Breast (0-IIIb) | RCT | Tai Chi instructor | 60 min | 3 |  | 12 weeks | Psychosocial support | 12 weeks | 31 | 68% | 100 |
| Campo 2013 2015 | US | Breast (I-III) | RCT | Tai Chi instructor | 60 min | 3 |  | 12 weeks | Health education control classes | 13 weeks | 54 | 86% | 100 |
| Irwin 2014 2017 | US | Breast (N.R.) | RCT | Tai Chi instructor | 40 min | 3 |  | 12 weeks | Cognitive behavioral therapy for insomnia | 15 months | 90 | 90% | 100 |
| Larkey 2015 2016 | US | Breast (0-III) | RCT | Nurse | 60 min | 2 |  | 2 weeks | Sham Qigong | 12 weeks | 87 | 80% | 100 |
|  |  |  |  |  |  | 1 |  | 10 weeks |  |  |  |  |  |
| Thongteratham 2015 | Thailand | Breast (0-IIIb) | RCT | Tai Chi instructor | 60 min | 3 |  | 12 weeks | Usual care | 12 weeks | 30 | 100% | 100 |
| Bonab 2020 | Iran | Breast (N.R.) | RCT | Tai Chi instructor | 60 min | 3 |  | 12 weeks | Usual care | 12 weeks | 30 | 100% | 100 |
| Fong 2014ab 2015 | HK, China | H&N (I-IV)^b^ | NRCT | Tai Chi instructor | 90 min | 1 | 3 | 70 weeks | Usual care | 70 weeks | 52 | 67% | 46.0 |
| a: RCT: randomized controlled trial, NRCT: non-randomized controlled trial  b: Head and neck cancer, nasopharyngeal carcinoma  c: Zhang 2013; Wang 2013; Liu 2015  d: Mustian 2004 2006 2008; Peppone 2010; Janelsins 2011; Sprod 2012 | | | | | | | | | | | | | |

| **Supplementary Table 4. Tai Chi forms and authors’ interpretation of Tai Chi of the included trials** | | | | | | | | | | | | | | | |
| --- | --- | --- | --- | --- | --- | --- | --- | --- | --- | --- | --- | --- | --- | --- | --- |
| **Study** | | **Tai Chi form** | **Author’s interpretation of Tai Chi** | | | | | | | | | | | | |
| **During chemotherapy** | | | |  | | | | | | | | | | | |
| Robins 2013 | | 8-forms Tai Chi | Complementary or “**mind-body**” interventions, including strategies for stress management. | | | | | | | | | | | | |
| Zhang 2016 | | 8-forms simplified Yang style Tai Chi | Traditional Chinese health-promoting exercise. Easily modifiable, low-to-moderate intensity form of **physical exercise**. | | | | | | | | | | | | |
| Ding 2020 | | 24-form Yang style Tai Chi | Tai chi is a traditional Chinese psychophysiological form of exercise which uses soft, slow, and coordinated movements to balance the strength of the limbs. There are different schools of tai chi, but they all share the same key characteristics, namely **mindfulness**, **structural adjustment**, and **flexibility**. | | | | | | | | | | | | |
| **During radiotherapy** | | | |  | | | | | | | | | | | |
| McQuade 2017 | | Based on Yang style, for cancer patients | The practices of qigong and tai chi have slow and gentle **movements**, combines physical postures or movements with focus on the **breath** and **mindfulness**. | | | | | | | | | | | | |
| **During chemoradiotherapy** | | | |  | | | | | | | | | | | |
| Zhou 2018 | | 24-form Yang style Tai Chi | **Mind-body exercise**: Tai Chi, a traditional oriental health-promoting exercise, is similar to aerobic exercise in several aspects. Besides low- to moderate-intensity physical activity, Tai Chi also contains elements of **meditation**, **body awareness**, and **breathing** | | | | | | | | | | | | |
| **Post-surgery** | |  |  | | | | | | | | | | | | |
| Zhang 2013; Wang 2013; Liu 2015 | | 24-form Yang style Tai Chi | Tai Chi is commonly described as “**meditation through movement**.” It incorporates elements of balance, postural alignment, and concentration. | | | | | | | | | | | | |
| Jiang 2020 | | 24-form Yang style Tai Chi | Tai Chi combines psychological intervention (mind wellbeing) and physical activities without special apparatus, and is widely practiced to promote pulmonary rehabilitation in China and increasingly learned in the world. **Mind-body** Tai Chi aims to control aerobic **activity**, **breathing** coordination, and **movement** techniques, which improves physical activity, dyspnea, and anxiety and depression, and is a potential approach in the improvement of pulmonary function. | | | | | | | | | | | | |
| **Post-mixed treatment** | | | | | | | | | | | | | | | |
| Mustian 2004 etc^a^ | | 15-form Yang style Tai Chi | Easily modifiable, low-to-moderate intensity form of **physical exercise** with psychological, physiological, and sociological benefits. | | | | | | | | | | | | |
| Campo 2013 2015 | | Tai Chi Chih | Tai Chi is a form of exercise that is also known as **meditative movement** because it **combines physical activity with meditation**; focused, fluid physical movements (i.e., balance and shifting of body weight), which are performed in a specified order, coordinated with **breathing** and imagery to relax the mind, strengthen the body, and improve the flow of “qi” or life energy. | | | | | | | | | | | | |
| Irwin 2014 2017 | | Tai Chi Chih | Tai Chi is a multidimensional, **mind–body** therapy that integrates moderate **physical activity**, deep **breathing**, and **meditation**, and has been found to offer a number of benefits in health and psychological functioning. | | | | | | | | | | | | |
| Larkey 2015 2016 | | Qigong/Tai Chi Easy | Qigong and Tai Chi are two forms of **Meditative Movement** with similar roots and practice components. Qigong translates from Chinese to mean, roughly, “to cultivate Qi”; and Qi is considered to be the inherent functional, energetic essence of human beings in Traditional Chinese Medicine. Qigong exercises consist of a series of simple, repeated practices including body posture/**movement**, **breath** practice, and **meditation** performed in synchrony. These exercises consist mostly of gentle movements (with some vigorous and shaking movements in addition to quiet, stillness practices) designed to attain deeply relaxed states. Tai Chi has become one of the best known forms of Qigong in the U.S., differentiated from Qigong in that traditional Tai Chi is performed as a highly choreographed, lengthy, and complex series of movements, while Qigong is typically a more repetitive practice that is simpler and easier to learn. | | | | | | | | | | | | |
| Thongteratham 2015 | | 18-forms Tai Chi Qi Qong | Tai Chi is a Chinese wisdom exercise that includes meditation and spiritual uplifting through simple but deep movements stimulating **body and mind** to induce relaxation. | | | | | | | | | | | | |
| Bonab 2020 | | Adapted 20-form Tai Chi | Tai Chi is an ancient Chinese martial art that uses gentle controlled **movement**, deep **breathing** and correct physical posture along with perceptual tools of vision, **focused inner consciousness** to strength the, relax the body and mind. | | | | | | | | | | | | |
| Fong 2014ab 2015 | | 18-forms Tai Chi Internal Qigong | Tai Chi and Qigong are ancient Chinese psychosomatic exercises comprising **meditation**, **breathing** exercises and coordinated **body movements**. | | | | | | | | | | | | |
| a: Mustian 2004 2006 2008; Peppone 2010; Janelsins 2011; Sprod 2012 | | | | | | | | | | | | | | | |
| **Supplementary Table 5a. Evidence level of common acute, long-term, and late effects of cancer reviewed for therapeutic efficacy of exercise^a^ that evaluated in Tai Chi interventions** | | | | | | | | | | | | | |  |  |
| Evidence level from exercise interventions | | | | Current evidence from Tai Chi interventions | | | | | | | | | |  |  |
| **Strong** | | | |  |  |  | Null | | Positive | | |  |  |  |  |
| Anxiety | | | |  |  |  |  |  |  |  |  |  |  |  |  |
| Depressive symptoms | | | |  |  |  |  |  |  |  |  |  |  |  |  |
| Fatigue | | | |  |  |  |  |  |  |  |  |  |  |  |  |
| Health related quality of life | | | |  |  |  |  |  |  |  |  |  |  |  |  |
| Lymphedema | | | |  |  |  |  |  |  |  |  |  |  |  |  |
| Physical function | | | |  |  |  |  |  |  |  |  |  |  |  |  |
| **Moderate** | | | |  |  | Null | | | Positive | | |  |  |  |  |
| Bone health | | | |  |  |  |  |  |  |  |  |  |  |  |  |
| Sleep | | | |  |  |  |  |  |  |  |  |  |  |  |  |
| **Insufficient** | | | |  |  | Null | | | Positive | | |  |  |  |  |
| Cardiotoxicity | | | |  |  |  |  |  |  |  |  |  |  |  |  |
| Chemotherapy-induced peripheral neuropathy | | | |  |  |  |  |  |  |  |  |  |  |  |  |
| Cognitive function | | | |  |  |  |  |  |  |  |  |  |  |  |  |
| Falls | | | |  |  |  |  |  |  |  |  |  |  |  |  |
| Nausea | | | |  |  |  |  |  |  |  |  |  |  |  |  |
| Pain | | | |  |  |  |  |  |  |  |  |  |  |  |  |
| Sexual function | | | |  |  |  |  |  |  |  |  |  |  |  |  |
| Treatment tolerance | | | |  |  |  |  |  |  |  |  |  |  |  |  |
|  | | | |  | | | | | | | | | |  |  |
| vs. nonactive control | | | | vs. active control | | | | | | | | | |  |  |
| a: In accordance with Compbell et al, Medicine & Sciences in Sports & Exercise 2019 | | | | | | | | | | | | | |  |  |

| **Supplementary Table 5b. Effect on common acute, long-term, and late effects of cancer of Tai Chi intervention** (↑ superior, ↔ equivalent/non-inferior, ↓ inferior) | | | | | | | | | | | | | |
| --- | --- | --- | --- | --- | --- | --- | --- | --- | --- | --- | --- | --- | --- |
| **Study** | **Cancer site (stage)** | **Control groups** | **Level of evidence from exercise interventions on common acute, long-term, and late effect of cancer** | | | | | | | | | | |
|  |  |  | **Strong** | | | | | | | **Moderate** | | **Insufficient** | |
|  |  |  | Anxiety | Depressive symptoms | | Fatigue | | Health  related QoL^a^ | Physical function | Bone health | Sleep | Cognitive function | Pain |
| **During chemotherapy** | |  |  | |  | |  |  |  |  |  |  |  |
| Robins 2013^b^ | Breast (early stage) | Nonactive |  | ↔ | |  | | ↔ |  |  |  |  |  |
|  |  | Active |  | ↔ | |  | | ↔ |  |  |  |  |  |
| Zhang 2016 | Lung (I-IV) | Active |  |  | | ↑ | |  |  |  |  |  |  |
| Ding 2020 | N.R. (N.R.) | Active |  |  | |  | | ↑ |  |  |  |  | ↔ |
| **During radiotherapy** | |  |  | |  | |  |  |  |  |  |  |  |
| McQuade 2017^b^ | Prostate (I-III) | Nonactive |  |  | | ↔ | |  |  |  | ↑ |  |  |
|  |  | Active |  |  | | ↔ | |  |  |  | ↑ |  |  |
| **During chemoradiotherapy** | |  |  | |  | |  |  |  |  |  |  |  |
| Zhou 2018 | H&N (III-IVa/b)^b^ | Nonactive |  |  | | ↑ | |  |  |  |  |  |  |
| **Post-surgery** | |  |  | |  | |  |  |  |  |  |  |  |
| Jiang 2020 | Lung (N.R.) | Active |  |  | |  | |  |  |  |  |  | ↑ |
| **Post-mixed treatment** | |  |  | |  | |  |  |  |  |  |  |  |
| Mustian 2004 etc^d^ | Breast (0-IIIb) | Active |  |  | | ↑ | | ↑ | ↑ | ↑ |  |  |  |
| Campo 2013 2015 | Breast (I-III) | Active |  |  | |  | | ↔ | ↔ |  |  |  |  |
| Irwin 2014 2017 | Breast (N.R.) | Active |  |  | |  | |  |  |  | ↔ |  |  |
| Larkey 2015 2016 | Breast (0-III) | Active |  | ↔ | | ↑ | |  | ↔ |  | ↔ | ↔ |  |
| Thongteratham 2015 | Breast (0-IIIb) | Nonactive |  |  | | ↑ | | ↑ |  |  |  |  |  |
| Bonab 2020 | Breast (N.R.) | Nonactive | ↑ | ↑ | |  | |  | ↑ |  | ↑ |  |  |
| Fong 2014ab 2015 | H&N (I-IV)^b^ | Nonactive |  |  | |  | | ↔ | ↔ |  | ↑ |  |  |
| a: Health-related quality of life  b: Trials included both active and nonactive (usual care or waitlist) control groups. Reporting effect estimates comparing Tai Chi vs. usual care group.  c: Head and neck cancer, nasopharyngeal carcinoma  d: Mustian 2004 2006 2008; Peppone 2010; Janelsins 2011; Sprod 2012 | | | | | | | | | | | | | |

| **Supplementary Table 6. Measures used to assess common acute, long-term, and late effects of cancer of Tai Chi intervention** | | | | | | | | | | | | | | |
| --- | --- | --- | --- | --- | --- | --- | --- | --- | --- | --- | --- | --- | --- | --- |
| **Study** | | **Cancer site (stage)** | | **Anxiety** | | **Depressive symptoms** | **Fatigue** | **Health**  **related QoL^a^** | **Physical function** | **Bone health** | | **Sleep** | **Cognitive function** | **Pain** |
| **During chemotherapy** | | | |  | |  |  |  |  |  | |  |  |  |
| Robins 2013 | | Breast (early stage) | |  | | CES-D |  | FACT-B |  |  | |  |  |  |
| Zhang 2016 | | Lung (I-IV) | |  | |  | MFSI-SF |  |  |  | |  |  |  |
| Ding 2020 | | N.R. (N.R.) | |  | |  |  | SF-36 |  |  | |  |  | SF-36 BP |
| **During radiotherapy** | | | |  | |  |  |  |  |  | |  |  |  |
| McQuade 2017 | | Prostate (I-III) | |  | |  | BFI |  |  |  | | PSQI |  |  |
| **During chemoradiotherapy** | | | |  | |  |  |  |  |  | |  |  |  |
| Zhou 2018 | | H&N (III-IVa/b)^b^ | |  | |  | MFSI-SF |  |  |  | |  |  |  |
| **Post-surgery** | | | |  | |  |  |  |  |  | |  |  |  |
| Jiang 2020 | | Lung (N.R.) | |  | |  |  |  |  |  | |  |  | VSA |
| **Post-mixed treatment** | | | |  | |  |  |  |  |  | |  |  |  |
| Mustian 2004 etc^c^ | | Breast (0-IIIb) | |  | |  | FACIT-F | SF-36 | SF-36 PF | Biomarker | |  |  |  |
| Campo 2013 2015 | | Breast (I-III) | |  | |  |  | SF-36 | SF-36 PF |  | |  |  |  |
| Irwin 2014 2017 | | Breast (N.R.) | |  | |  |  |  |  |  | | PSQI |  |  |
| Larkey 2015 2016 | | Breast (0-III) | |  | | BDI | FSI |  | SF-36 PF |  | | PSQI | FACT-COG |  |
| Thongteratham 2015 | | Breast (0-IIIb) | |  | |  | FSI | FACT-G |  |  | |  |  |  |
| Bonab 2020 | | Breast (N.R.) | | GHQ | | GHQ |  |  | GHQ |  | | GHQ |  |  |
| Fong 2014ab 2015 | | H&N (I-IV)^b^ | |  | |  |  | EORTC QLQ-C30 | QLQ-C30 PF |  | | MOS-Sleep |  |  |
| a: Health-related quality of life  b: Head and neck cancer, nasopharyngeal carcinoma  c: Mustian 2004 2006 2008; Peppone 2010; Janelsins 2011; Sprod 2012  **Abbreviations of outcome measures:**  CES-D: Center for Epidemiological Studies Depression Scale  FACT-B: Functional Assessment of Cancer Therapy-Breast cancer  MFSI-SF: Multidimensional Fatigue Symptom Inventory-Short From  SF-36: 36-Item Short Form Health Survey  SF-36 BP: 36-Item Short Form Health Survey Bodily Pain  BFI: Brief Fatigue Inventory  PSQI: Pittsburgh Sleep Quality Index  VSA: Visual Analog Scale for Pain  FACIT-F: Functional Assessment of Chronic Illness Therapy-Fatigue  SF-36 PF: 36-Item Short Form Health Survey Physical Functioning  BDI: Beck Depression Inventory  FSI: Fatigue Symptom Inventory  FACT-COG: Functional Assessment of Cancer Therapy-Cognitive Function  FACT-G: Functional Assessment of Cancer Therapy-General  GHQ: General Health Questionnaire (subscales: physical, anxiety and insomnia, social dysfunction, depression)  EORTC QLQ-C30: The European Organization for Research and Treatment of Cancer Quality of Life Questionnaire  QLQ-C30 PF: The European Organization for Research and Treatment of Cancer Quality of Life Questionnaire Physical Function  MOS-Sleep: The Medical Outcomes Study Sleep Scale | | | | | | | | | | | | | | |
| **Supplementary Table 7. Other cancer related outcomes of Tai Chi intervention** (↑ superior, ↔ equivalent/non-inferior, ↓ inferior) | | | | | | | | | | | | | | |
| **Study** | **Cancer site (stage)** | | **Control groups** | | **Other outcomes** | | | | | | | | | |
|  |  |  |  |  | **Biomarkers** | | | | | | **Non-biological outcomes** | | | |
| **During chemotherapy** | | |  | |  | | | | | |  | | | |
| Robins 2013^a^ | Breast (early stage) | | Nonactive | | Neuroendocrine (↔)  Immunological measures (↑IL-1𝛽, IFN-𝛾) | | | | | | Stress (↔) | | | |
|  |  | | Active | | Neuroendocrine (↔)  Immunological measures (↔) | | | | | | Stress (↔) | | | |
| Ding 2020 | N.R. (N.R.) | | Active | |  | | | | | | Self-management ability (↑)  Exercise ability (↑)  Incidence of venous thrombosis (↑)  Coagulation function index (↑) | | | |
| **During radiotherapy** | | |  | |  | | | | | |  | | | |
| McQuade 2017^a^ | Prostate (I-III) | | Nonactive | |  | | | | | | Hormonal function (↓) | | | |
|  |  | | Active | |  | | | | | | Hormonal function (↔) | | | |
| **During chemoradiotherapy** | | |  | |  | | | | | |  | | | |
| Zhou 2018 | H&N (III-IVa/b)^b^ | | Nonactive | |  | | | | | | Heart rate variability (↑) | | | |
| **Post-surgery** |  | |  | |  | | | | | |  | | | |
| Zhang 2013; Wang 2013; Liu 2015 | Lung (I-IIIb) | | Nonactive | | Cellular immunity (CD55 ↑)  Cellular and humoral immunity balance (↑)  Peripheral blood mononuclear cells proliferative and cytolytic activities (↑) | | | | | |  | | | |
| Jiang 2020 | Lung (N.R.) | | Active | | Antioxidant makers (↑)  Inflammatory markers (↑)  Blood oxygen level (↑) | | | | | | Lung function (↑) | | | |
| **Post-mixed treatment** | | |  | |  | | | | | |  | | | |
| Mustian 2004 etc^c^ | Breast (0-IIIb) | | Active | | Inflammatory cytokines and insulin (↑) | | | | | | Self-esteem (↑)  Body composition (↔)  Aerobic capacity (↑)  Handgrip strength (↑) | | | |
| Campo 2013 2015 | Breast (I-III) | | Active | | Inflammatory cytokines (↔)  Salivary cortisol (↑) | | | | | | Mental health related quality of life (↑)  Blood pressure (↑) | | | |
| Irwin 2014 2017 | Breast (N.R.) | | Active | | Systematic inflammatory markers (↔)  Cellular inflammatory markers (↑)  Genomic inflammatory markers (↑) | | | | | |  | | | |
| Larkey 2015 2016 | Breast (0-III) | | Active | |  | | | | | | Body mass index (↑)  Self-reported physical activity (↔) | | | |
| Thongteratham 2015 | Breast (0-IIIb) | | Nonactive | | Urine Free Cortisol (↑) | | | | | | Self-esteem (↑) | | | |
| Bonab 2020 | Breast (N.R.) | | Nonactive | |  | | | | | | Social dysfunction (↑) | | | |
| Fong 2014ab 2015 | H&N (I-IV)^b^ | | Nonactive | |  | | | | | | Cervical side flexion (↑)  Functional aerobic capacity (↑) | | | |
| a: Trials included both active and nonactive (usual care) control groups. Reporting effect estimates comparing Tai Chi vs. usual care group.  b: Head and neck cancer, nasopharyngeal carcinoma  c: Mustian 2004 2006 2008; Peppone 2010; Janelsins 2011; Sprod 2012 | | | | | | | | | | | | | | |

**Supplementary Table 8.** Summary of study quality assessment based on the Risk of Bias 2 tool

Supplementary Figure 1: Study flowchart

Records identified through

database searching (n=1755)

Records screened after duplicates removed (n=797)

Full text articles excluded (n=27):

Focused solely on Qigong (n=21)

No control group (n=2)

No quantitative outcomes assessed (n=1)

Case study and small sample size (n=2)

Single bout Tai Chi intervention (n=1)

Full text articles assessed for eligibility (n=53)

Studies included (n=26)

Additional records identified through other sources (n=0)

Records excluded (n=744)

**Detailed description of each included trials of study design and findings**

*Tai Chi interventions during active treatment*

Five Tai Chi trials were conducted during active cancer treatment including chemotherapy: breast, lung and mixed cancer; radiotherapy: prostate cancer; and chemoradiotherapy: nasopharyngeal carcinoma.

Robins et al^30^ randomized 145 women with early-stage breast cancer undergoing adjuvant chemotherapy into one of three groups: 8-form Tai Chi training, spiritual growth, and usual care as control. The Tai Chi training consisted of weekly 90 minutes supervised session. Each of the Tai Chi and spiritual growth interventions were 10 weeks, and participants were followed up at three, four and half and six months from the baseline. With a total retention rate of 75%, authors reported no significant difference between the three groups for depression symptoms, quality of life or stress. Nevertheless, both Tai Chi and spiritual groups appeared to improve immunological biomarkers (Interleukin-1𝛽 [IL-1𝛽] and interferon gamma [IFN-𝛾]) comparing to usual care.

Zhang et al^44^ randomized 96 lung cancer patients to 8-form simplified Yang style Tai Chi intervention or a low-impact exercise group for 12 weeks through four cycles of chemotherapy. Tai Chi and low-impact exercise started from the 10^th^ day during each of the 21-day chemotherapy cycle. Both Tai Chi training and low-impact exercise consist of 60 minutes session every other day in the morning either in the community led by experienced Tai Chi instructors or at home by instructional DVD. Cancer-related fatigue were assessed at baseline, end of 2^nd^ (85% retention) and end of the 4^th^ (77% retention) chemotherapy cycle, and that was better managed in the Tai Chi group with lower general fatigue, physical fatigue and higher vigor comparing to the low-impact exercise group.

Another Tai Chi intervention during chemotherapy was recently completed by Ding et al,^45^ which randomised 98 lung cancer patients to either a traditional handgrip exercise group or a 24 form-simplified Yang style Tai Chi group for 12 weeks (retention 100%). The Tai Chi exercise was at least 60 minutes per session that was initially supervised by a coach then self-led by patients on at least five days per week, while the handgrip exercise was done by giving patients a grip ball and detailed daily training schedule. At the end of the intervention, Tai Chi improved quality of life, but not pain, comparing to handgrip exercise. Notably, Tai Chi group also shown improvement on self-management ability, exercise ability, incident varooms thrombosis, and coagulation function index comparing to handgrip exercise.

Only one Tai Chi intervention was conducted during radiotherapy. McQuade et al^31^ randomized 76 prostate cancer patients to either a group of Tai Chi exercise for cancer patients based on the Yang style, a light exercise group or waitlist control group for seven-eight weeks throughout their radiotherapy. Both Tai Chi and light exercise groups consist of three times weekly 40 minutes supervised sessions with recommended daily practise at home. Participants were followed up at end of the treatment (82% retention rate), four-week (66% retention) and 12-week (66% retention) after the treatment. Tai Chi improved sleep midway of the intervention comparing to light exercise and waitlist control, and that no difference was observed for fatigue between groups. Also, Tai Chi and light exercise both improved self-reported hormonal function comparing to waitlist control.

Finally, Zhou et al^46^ conducted a Tai Chi intervention during chemoradiotherapy, that randomized 114 patients with nasopharyngeal carcinoma to either a 19-week simplified 24-form Yang style Tai Chi group or usual care control group. Tai Chi exercise was done at five time per week of 60 minutes supervised session and participants were followed up by the end of the intervention (73% retention). Intention-to-treat and per-protocol analyses both showed improved fatigue and heart rate variability in the Tai Chi group comparing to control.

*Tai Chi interventions post-surgery*

Two trials from four papers were conducted after surgery for resectable non-small cell lung cancer. Three papers^47-49^ reported finding from the same trial, which randomized 32 post-surgical non-small cell lung cancer survivors into either a 16-week 24-form Yang style Tai Chi group or usual care control group. Tai Chi exercise was done at three times per week of 60 minutes supervised session and participants were followed up by the end of the intervention (84% retention). This trial was designed to evaluate the biological mechanisms of Tai Chi, and all study outcomes were derived from the serum sample. This trial found that Tai Chi improved biomarkers of cellular immunity,^47^ cellular and hormonal immunity balance,^48^ and peripheral blood mononuclear cells proliferative and cytolytic activities.^49^

A more recent trial done by Jiang et al^50^ randomized 100 post-surgical non-small cell lung cancer survivors into either a 12-week 24-form Yang style Tai Chi group or common care physical exercise group. Tai Chi exercise was done daily 60 minutes supervised session in the morning, and the common care physical exercise was done in equal intensity of duration to Tai Chi using exercise calorie calculators. Participants were followed up by the end of the intervention (100% retention). This trial found that Tai Chi improved patient reported pain, lung function, increased antioxidant and blood oxygen level, and reduced inflammatory markers comparing to physical exercise.

*Tai Chi interventions post mixed treatment modalities*

Sixteen reports from seven trials were conducted among post-treatment cancer survivors of mixed treatment modalities: six in breast and one in nasopharyngeal carcinoma.

Four trials conducted by Mustian et al (n=31, retention 68%),^32-37^ Campo et al (n=63, retention 86%),^38, 39^ Irwin et al (n=90, retention 90%)^40, 41^ and Larkey et al (n=101, retention 80%)^42, 43^ all enrolled breast cancer survivors and randomized them into either a 12-week Tai Chi intervention or an active control group, including psychosocial support, health education control classes, cognitive behavioral therapy for insomnia, and Sham Qigong, respectively. Among these studies, Mustain et al^32-37^ used Yang style Tai Chi, Compo et al^38, 39^ and Irwin et al^40, 41^ used Tai Chi Chih, and Larkey et al^42, 43^ used Qigong/Tai Chi easy. The Tai Chi intervention was three time per week of 60 minutes supervised session in Mastian et al^32-37^ and Compo et al, ^38, 39^ once per week 120 minutes supervised session in Irwin et al, ^40, 41^ and twice per week for the first two week then once per week for the remaining ten weeks of 60 minutes supervised sessions in Larkey et al.^42, 43^ The follow-up time varied between 12 weeks (at the end of the intervention) to 15 months from baseline. These trials observed improvements in fatigue, quality of life in one trial, bone health comparing to their respective controls and no group difference in depressive symptoms, self-reported physical function, sleep, and cognitive function. In addition, studies that assessed biological markers showed that Tai Chi improved inflammatory cytokines and insulin comparing to psychosocial support, improved salivary cortisol but not inflammatory cytokines when comparing to health educating control class. Other outcomes assessed that were improved in Tai Chi vs. control were aerobic capacity, handgrip strength, mental health related quality of life, self-esteem, blood pressure and body mass index. Notably, Irwin et al^41^ was a noninferiority trial showing that Tai Chi was noninferior to the gold standard of behavioral treatment of insomnia (cognitive behavioral therapy). The secondary analyses of the same trial^40^ showed that Tai Chi improved cellular and genomic inflammatory makers but not systematic inflammatory maker comparing to cognitive behavioral therapy.

The other two Tai Chi interventions in breast cancer survivors were done by Thongteratham et al (n=30, retention 100%)^54^ and Bonab et al (n=30, retention 100%),^55^ which compared a 12-week Tai Chi intervention to usual care controls. The Tai Chi intervention were three times per week of 60 minutes supervised session in both trials, while the 18-form Tai Chi Qi Gong and Kata format Tai Chi were used in Thongteratham et al^54^ and Bonab et al,^55^ respectively. These two trials follow-up participants by the end of the 12-week intervention, and reported improvement in patient reported anxiety, depressive symptoms, fatigue, health related quality of life, physical function and sleep. These two trails also reported improvements in patient reported self-esteem, social dysfunction and urine free cortisol.

Finally, Fong et al^51-53^ conducted a non-randomized control trial that assigned 25 participants with nasopharyngeal carcinoma to a 70-week (6 months) 18-form Tai Chi internal Qigong based on their willingness and enrolled 27 participants in the usual control group. The Tai Chi exercise was done at once per week of 90 minutes supervised session and three time per week of 90 minutes unsupervised session. At the end of the intervention, the overall retention was 67%. Intention-to-treat analyses suggested improved sleep and no between group difference in health-related quality of life or physical function. Nevertheless, improved cervical side flexion and functional aerobic capacity were reported in the Tai Chi group comparing to usual care
